# Structural characteristics of polysaccharide from *Zingiber striolatum* and its effects on gut microbiota composition in obese mice

**DOI:** 10.3389/fnut.2022.1012030

**Published:** 2022-10-26

**Authors:** Wei Jiang, Ying Hu, Zhenyuan Zhu

**Affiliations:** ^1^Key Laboratory of Food Nutrition and Safety, Ministry of Education, Tianjin Key Laboratory of Food Nutrition and Safety, Tianjin University of Science and Technology, Tianjin, China; ^2^College of Food Science and Engineering, Tianjin University of Science and Technology, Tianjin, China; ^3^Department of Health Management, Zunyi Medical and Pharmaceutical College, Guizhou, China; ^4^School of Public Health, Zunyi Medical University, Guizhou, China

**Keywords:** *Zingiber strioatum*, polysaccharide, chemical structure, gut microbiota, obesity

## Abstract

To investigate a polysaccharide from *Zingiber striolatum* favorably modulates gut microbiota in mice fed a high-fat diet. *Z. striolatum* was utilized to extract the crude polysaccharide CZSP, which was subsequently refined using DEAE-52 cellulose and Sephadex G-150 to yield the novel polysaccharide *Zingiber strioatum* pure polysaccharide-1 (ZSPP-1). ZSPP-1 was an acidic heteroglycan made up of galactose, mannose, glucose, xylose, arabinose, glucuronic acid, and galacturonic acid with an average molecular weight of 1.57 × 10^6^ Da. The structure of ZSPP-1 was investigated by FT-IR, methylation and NMR analysis, and the results denoted that the linkage structure types include T-Man*p*-linked, β-Xyl*p*-(1,2)-linked, β-Gal*p*-(1,4)-linked, α-Glc*p*A-(1,6)-linked, β-Ara*p*-(1,4)-linked, α-Glc*p*-(1,3,4,6)-linked, α-Glc*p*-(1,2)-linked, and β-T-Xyl*p*-linked, in which β-Gal*p*-(1,4)-linked and α-Gal*p*A-(1,4)-linked might be the main linkage. The results of the intervention experiments showed that ZSPP-1 changed the intestinal flora structure of the Firmicutes and Bacteroidetes in obese mice, and promoted the growth of beneficial bacteria such as *Akkermansia*, *Lactobacillus*, and *Bacteroides* in the intestine. It also restored the imbalanced flora structure due to high-fat diet to normal. It also restored the imbalanced flora structure due to high-fat diet to normal. *Z. striolatum* polysaccharides presented a considerable advantage in alleviating high-fat diet induced obesity, which indicates that it can be further exploited as a natural functional food resource.

## Introduction

Intestinal microbes are crucial for digestion and absorption of food; However, research indicates that intestinal microbial imbalance is intimately linked to obesity and related chronic metabolic illnesses (1). Since Fredrik Bäckhed et al. (2) initially postulated in the correlation between obesity and gut microbiota (i.e., It is confirmed that gut microbiota, as an environmental factor, regulates the accumulation of fat in the host). Zou et al. (3) transplanted the intestinal flora of obese and lean mice into new host mice; the results demonstrated that the new host mice had identical fat and lean phenotypes as the donor. Therefore, the tight association between obesity and intestinal microflora has drew the attention of an increasing number of researchers. Firmicutes/Bacteroidetes (F/B value) in the composition of intestinal microflora is a typical biomarker of intestinal microflora imbalance; the decrease or increase of F/B value indicates the remission of obesity or fat accumulation (4). Intestinal flora serves as a biological barrier to protect the body from hazardous toxins (such as endotoxin, harmful bacteria, etc.). Once intestinal flora is out of equilibrium, the gut wall becomes more permeable, allowing hazardous substances to invade the body. Obesity usually is accompanied by inflammation, and the increase of lipopolysaccharide in blood aggravates the inflammatory response (5). Obesity and the associated metabolic syndrome could be alleviated by probiotic supplementation (6). Consequently, the restoration of gut microbiota balance is regarded as a crucial intervention target for the prevention and treatment of obesity and related chronic metabolic illnesses (7).

Intestinal microbes are highly capable of digesting polysaccharides and can interact with intestinal epithelial cells, hence influencing energy intake and obesity (8). Plant polysaccharides are natural products extracted from plants; although the absorption efficiency of plant polysaccharides in the mammalian gastrointestinal tract is very low, studies have exhibited that the majority of them have a wide range of pharmacological effects, particularly the regulation of glycolipid and energy metabolism, which is often believed to be closely related to the regulation of gut microbiota (9).

*Zingiber striolatum* (*Zingiber striolatum Diels*) is a perennial herb belonging to *Zingiberaceae* which has certain special biological activities and can be used as a potential plant polysaccharide resource for the regulation of gut microbiota *Z. striolatum* is frequently utilized for vegetable consumption or traditional Chinese medicine (i.e., It is principally for the treatment of diseases including constipation, diabetes, hypertension, hyperlipidemia and inflammation). As a member of plant polysaccharides, the polysaccharide from *Z. striolatum* also has a variety of physical and chemical properties. However, little is known regarding the precise chemical structure and effect on glycolipid metabolism *in vivo* of the polysaccharides purified from *Z. striolatum*.

In this study, a novel acidic polysaccharide termed ZSPP-1 was purified from *Z. striolatum*. SEM imagery, molecular mass, physicochemical characterization, monosaccharide composition, Fourier transform infrared spectrometer (FT-IR) spectrogram, methylation, and 1D-NMR and 2D-NMR analyses were performed to characterize ZSPP-1. In the meantime, the effect of ZSPP-1 on the gut microbiota of obese mice was also investigated, which could provide theoretical reference for the clinical application and development of the polysaccharide.

## Materials and methods

### Materials and chemical reagents

Fresh *Z. striolatum* were harvested in Qixingguan County, Bijie City, Guizhou Province, China. All chemicals and solvents utilized in the study were analytical reagent grades. 20–22 g male SPF C57BL/6J mice, 6–8 weeks old, production license number: SCXK (Beijing, 2019-0010) were supplied by Sibeifu Biotechnology Co., Ltd. (Beijing, China). Complying with the pertinent legislation and the Guide for the Care and Use of Laboratory Animals, animal welfare and experimental procedures were conducted [Ministry of Science and Technology of China, (10)]. The license number of the experimental unit is SYXK (Tianjin) 2018-0001. None of the experimentation involved human subjects.

### Extraction of crude polysaccharide of *Zingiber striolatum*

*Zingiber striolatum* were rinsed in water to remove soil from their surface, then drained and air-dried. Afterward, *Z. striolatum* were cut into pieces, dried and crushed into powder (100 mesh). The powder was then rehydrated 20 times with water, thoroughly mixed, extracted for 2 h in an 80°C water bath, and filtered with a brinell funnel to secure filtrate. Next, the filter residues were extracted twice and all filtrates were combined. Filtrates were concentrated at 60°C under reduced pressure, then precipitated overnight at 4°C with four times the volume of 95% ethanol. The precipitates were collected after centrifugation at 4,000 r⋅min^–1^ for 10 min. The precipitate is dissolved in an adequate volume of distilled water, placed in the cone separation funnel, thoroughly mixed with 1/4 volume of sevage reagent, and allowed to stand for 4 h. After stratification, the mixed layer of sevage and protein in the cone separation funnel’s intermediate and lower layers is removed and the process is repeated numerous times. Evaporate and concentrate the crude protein-free polysaccharide solution to evaporate the remaining sevage reagent in the polysaccharide solution. Last but not least, there is a large amount of pigment in *Z. striolatum*, 1/2 volume of AB-8 macroporous adsorption resins are added to the concentrated solution and placed in a shaking table for 160 rpm overnight, in order to remove the pigment from the polysaccharide. The crude polysaccharide of *Z. striolatum* (CZSP) was eventually obtained after the polysaccharide was placed in a 100 kDa dialysis bag, dialyzed with flowing water and distilled water for 48 h respectively, and then vacuum freeze-dried.

### Purification of crude polysaccharide of *Zingiber striolatum*

Crude polysaccharide of *Z. striolatum* was applied to a DEAE Cellulose-52 column and stepwise elution with distilled water (0.1, 0.3, and 0.5 M NaCl) at a flow rate of 1.2 mL/min was then conducted to produce four fractions (ZSP-1, ZSP-2, and ZSP-3). The principal fraction (ZSP-1) was subsequently desalted by filtration through membranes with a molecular mass of 3,500 Da. CZSP-1 was preserved following freeze-drying under vacuum. Then 10 mg of ZSP-1 was redissolved in 1 mL of deionized water and filtered through a 0.45 m membrane. Sephadex G-150 was utilized for separation, while distilled water was employed for elution. Before elution, it was allowed to stand and balance for 10 min, the elution flow rate was 0.16 mL/min, and 1.3 mL of eluent is gathered from each collecting tube. At 490 nm, the sugar content of the eluent was measured employing the phenol sulfuric acid methodology. To acquire *Z. striolatum* pure polysaccharide, also known as ZSPP-1, the elution curve was drawn, the major peak eluent was collected, the identical components were combined, and lyophilization was used.

### Characterization of ZSPP-1

#### Scanning electron microscopy analysis

With an SU1510 electron microscope, SEM image of lyophilized ZSPP-1 were observed (Hitachi, Japan). Prior to measurements, the specimens’ surfaces were coated with a thin gold film to optimize conductivity.

#### Homogeneity and molecular mass determination

Homogeneity and molecular mass of ZSPP-1 were determined employing high performance liquid chromatography with an Agilent 1200 HPLC system outfitted with a TSK-GEL G4000PWxl column and a refractive index detector (RID). Sample (10 μL) solution (1 mg/mL) was injected in each run, with ultrapure water as the mobile phase at a flow rate of 0.6 mL/min (30°C). The molecular mass of ZSPP-1 was evaluated by comparing with the retention times based on the standard curve of a succession of molecular mass standards (10, 40, 70, 500, and 2,000 kDa) (11).

#### Physicochemical characterization determination

The total carbohydrate content was measured by the phenol sulfuric acid methodology using D-glucose as the benchmark. At 490 nm, the absorbance was observed, and the glucose standard curve was constructed (12). With galacturonic acid as the reference, the content of uronic acid was evaluated by sulfuric acid carbazole methodology, the absorbance was detected at 525 nm, and the galacturonic acid standard curve was constructed (13). The protein content was assessed using the Coomassie brilliant blue G-250 method, with bovine serum protein serving as the standard, and the absorbance was measured at 595 nm. Furthermore, the protein content of *Z. striolatum* polysaccharide was validated by the UV absorption spectrum recorded between 190 and 400 nm using a spectrophotometer.

#### Monosaccharide composition determination

The monosaccharide composition of ZSPP-1 was evaluated using gas chromatography-mass spectrometer (GC-MS) with some modifications (14). Add 1.5 mL of a 2 mol/L TFA solution to a ZSPP-1 sample (10 mg) and hydrolyze it in an oil bath heated to 110°C for 3 h. After hydrolysis was complete, dry with nitrogen and add 1 ml of distilled water to generate ZSPP-1 hydrolysis solution. ZSPP-1 hydrolysis solution was mixed with 0.5 mol/L sodium carbonate solution (reacting in a 30°C water bath for 30 min), 0.5 mL 4% sodium borohydride solution (reacting in a 30°C water bath for 1.5 h) was then added, and 25% acetic acid solution is dropped until no bubbles emerge. The reaction solution was eluted with distilled water after passing through the cation exchange column Dowex-50w8-200 (type H+). Following collecting the eluent, evaporate the sample under reduced pressure at 80°C until it is completely dry. The dry residue added 1 mL of pyridine and n-propylamine, respectively (sealed and dried with nitrogen after reacting at 55°C for 30 min), and added 2 mL of pyridine and acetic anhydride, respectively (reacted overnight at 25°C, dried with nitrogen). The residue is diluted in 1 mL of carbon dichloride, extracted with water for 2–3 times to eliminate impurities, and the organic solvent layer is filtered through a 0.22 μm membrane for GC-MS analysis.

#### Fourier transform infrared spectrometer analyses

The structure of ZSPP-1 was investigated by FT-IR employing a Vector 22 FT-IR (Bruker, Germany) operated in the region of 400–4,000 cm^–1^. The infrared spectra were gathered at resolution of 2 cm^–1^ with 16 scans. ZSPP-1 were separately ground with KBr powder and compressed into pellets for FT-IR measurement. Briefly, the samples (1 mg) and KBr (150 mg) were precisely weighed and compressed into pellets, and the data was collected with the FT-IR.

#### Methylation analysis

The methylation experimental method was referenced and slightly modified (15). Before methylation, acidic polysaccharide uronic acid should be decreased. Initially, 5 mg of ZSPP-1 was dissolved in 5 mL water. Afterward, 5 mg of carbodiimide was added to the ZSPP-1 acidic polysaccharide solution, and the pH was adjusted to 4.75 using 0.1 M hydrochloric acid (stirring evenly). Then, 5 mL of sodium boron deuterate solution (while stirring constantly at pH = 7.0 for 0.5 h) was added to the solution and the pH was adjusted to 4.0 using 2 M hydrochloric acid. The reaction solution was dialyzed overnight with 3,500 Da at 25°C. The Reduced sample was dried and set it aside. The sample was added 2 mL mixed solution of acetic acid and methanol (v/v = 1/9), dried with nitrogen and repeated for four times. Finally, the sample was added a few drops of methanol and dried the solution with nitrogen to eliminate any residual boric acid.

The dry neutral ZSPP-1 was dissolved in 2 mL dimethyl sulfoxide (DMSO with an anhydration with 3A molecular sieves). Under nitrogen protection, 25 mg NaH was added to the solution (reacted in ultrasonic and darkened settings at 18–20°C for 30 min). Then, 1 mL of iodomethane was added to the solution, and the reaction was maintained at the same conditions for 1 h. Repeat these methylation operations more than five times, and add 0.5 mL of water to terminate the reaction. The methylation sample was extracted using carbon dichloride and dried with nitrogen. The methylation sample was then evaluated using FT-IR.

The methylation sample was hydrolyzed with 2 mL trifluoroacetic acid (2 mol/L) at 110°C for 3 h. The hydrolyzate was dried with nitrogen and added 25 mg sodium boron deuterate with 2 mL deionized water (reacted at 25°C for 2 h). The solution was pH-adjusted to 5.0 with ethylic acid and then nitrogen-dried. The dried sample was co-distilled with 3 mL of methanol and 0.1 mL of acetic acid five times in order to decompose the sodium boron deuterate (no ethylic acid at last two times). Next, ZSPP-1 polysaccharide derivatives need to be prepared. Reduced methylated sample were dissolved in 2 mL of acetic anhydride (reacted at 100°C for 1 h) and dried with nitrogen. Following that, the residue was co-distilled to dry with 3 mL methanol (three times) and dissolved with 1 mL dichloromethane for GC-MS analysis.

#### NMR spectroscopy analyses

The 1D (1H, 13C) and 2D (HSQC) NMR spectra were investigated by a Bruker AVANCE III HD 600 MHz spectrometer (Brucker, Germany). Each sample (50 mg) was dissolved in D_2_O (0.5 mL) at 25°C, and the data was analyzed employing Bruker Topspin-NMR software.

#### Animal experiment design

Sixty mice (6 weeks old) were classified into six groups. All mice were quarantined at the Tianjin University of Science and Technology Experimental Animal Center before being placed in the experimental chamber at a temperature (21–24°C), humidity (50–60%), and a 12 h light/dark cycle. The C57BL/6J mice were fed normally for a week. Then, nine mice were chosen at random to constitute the normal group (NG), which continued to receive basal fodder.

In comparison to the average weight of the NG group, the average weight of mice fed a high-fat diet for roughly 9 weeks was significantly greater than that of the NG group. When mice with a weight of over 20% were identified as obese mice, denoting that the obesity model was successfully constructed. The NG group continued to be fed basal fodder. The obese mice with successful modeling were randomly divided into five subgroups: the model group (MG) was fed with HFD and given the same amount of distilled water as ZSPP-1 every day; The low-dose group (LZG), medium-dose group (MZG), and high-dose group (HZG) were fed with HFD and given ZSPP-1 polysaccharide of 100, 200, and 400 mg/kg for at least 7 weeks, respectively; The 100 mg/kg Orlistat was utilized to gavage mice as positive control group (CG). The body weight of mice in each treatment group was recorded every week.

#### Fecal sample collection

Before the mice were sacrificed, the anal region was cleaned with medical alcohol, the tail was fixed, and the lower abdomen was stimulated with cotton swabs to induce defecation. When mice defecated, sterilized EP tubes were used to collect the feces. Following the collection of around eight feces from each mouse, the EP tubes were placed in the −80°C refrigerator for sequencing of gut microbiota.

#### Intestinal flora analysis

##### DNA extraction and PCR amplification

Microbial community genomic DNA was extracted from mice feces samples using the E.Z.N.A.^®^ soil DNA Kit (Omega Bio-tek, Norcross, GA, USA) according to manufacturer’s instructions. The DNA extract was checked on 1% agarose gel, and DNA concentration and purity were investigated with NanoDrop 2000 UV-vis spectrophotometer (Thermo Scientific, Wilmington, DE, USA). The hypervariable region V3–V4 of the bacterial 16S rRNA gene were amplified with primer pairs 338F (5′-ACTCCTACGGGAGGCAGCAG-3′) and 806R (5′-GGACTACHVGGGTWTCTAAT-3′) by an ABI GeneAmp^®^ 9700 PCR thermocycler (ABI, Los Angeles, CA, USA). The PCR amplification of 16S rRNA gene was implemented as follows: initial denaturation at 95°C for 3 min, followed by 27 cycles of denaturing at 95°C for 30 s, annealing at 55°C for 30 s and extension at 72°C for 45 s, and single extension at 72°C for 10 min, and end at 4°C. The PCR mixtures contain 5 × TransStart FastPfu buffer 4 μL, 2.5 mM dNTPs 2 μL, forward primer (5 μM) 0.8 μL, reverse primer (5 μM) 0.8 μL, TransStart FastPfu DNA Polymerase 0.4 μL, template DNA 10 ng, and finally ddH_2_O up to 20 μL. PCR reactions were conducted in triplicate. The PCR product was extracted from a 2% agarose gel, purified utilizing the AxyPrep DNA Gel Extraction Kit (Axygen Biosciences, Union City, CA, USA) in accordance with the manufacturer’s instructions, and quantified by Quantus™ Fluorometer (Promega, USA).

##### Illumina MiSeq sequencing

Purified amplicons were pooled in equimolar and paired-end sequenced on an NovaSeq PE250 platform in accordance with the standard protocols by Majorbio Bio-Pharm Technology Co., Ltd. (Shanghai, China).

##### Processing of sequencing data

The raw 16S rRNA gene sequencing reads were demultiplexed, filtered for quality by fastp version 0.20.0 (16) and merged by FLASH version 1.2.7. Using UPARSE version 7.1, operational taxonomic units (OTUs) with a similarity cutoff of 97% were clustered, and chimeric sequences were spotted and eliminated. RDP Classifier version 2.2 was used to examine the taxonomy of each OTU representative sequence (17) against the 16S rRNA database using confidence threshold of 0.7.

##### Upload the data to the repository

The original sequencing data is uploaded to the official NCBI website. Link to: https://submit.ncbi.nlm.nih.gov/subs/. Registration No.: PRJNA871034.

#### Statistical analysis

The data were expressed as mean ± standard deviation (SD), where the mean data was calculated using Excel, 2010. SPSS 22 software was employed for one-way ANOVA and Duncan test for significance analysis, and the *P*-value < 0.05 was determined to be statistically significant.

## Results and discussion

### The SEM images and physicochemical compositions of ZSPP-1

**FIGURE 1:**
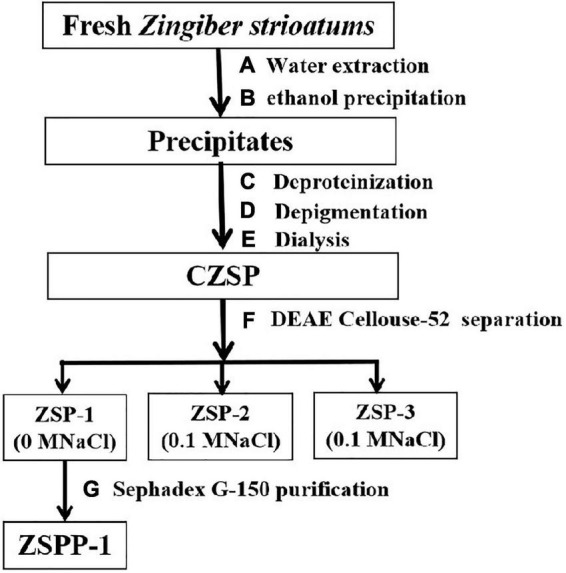
The procedure of ZSPP-1 extraction and purification.

**FIGURE 2:**
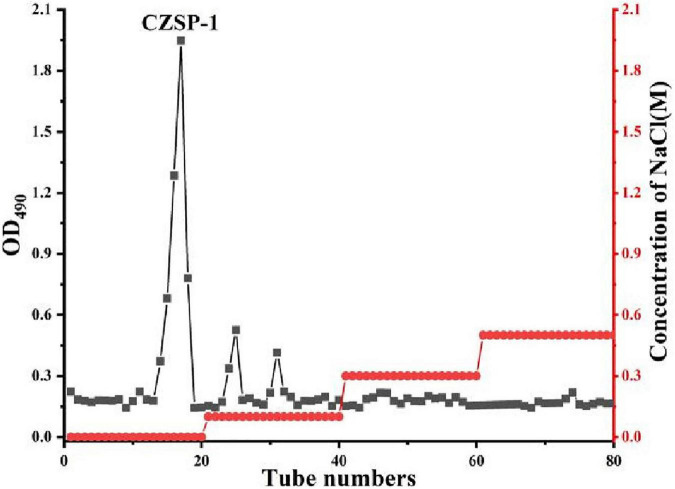
Elution profile of CZSP-1 on DEAE-cellulose-52 column.

The separation and purification flow chart of CZSP illustrated in Figure 1. CZSP is separated into three parts on DEAE-cellouse-52 column (Figure 2). As shown in the figure, ZSP-1 is higher in absorbance compared with ZSP-2 and ZSP-3. It can also be stated that ZSP-1 produced a better yield. In this study, only ZSP-1 that has been purified by Sephadex G-150 will be examined; the other two components will be used in other studies. The ZSPP-1 was obtained as depicted in Supplementary Figure 1. The percentages of total sugar, uronic acid, and protein in ZSPP-1 were approximately 90.6%, 12.31%, and undetectable, respectively. UV absorption spectrum further revealed that ZSPP-1 lacked absorption peaks at 260 and 280 nm (Supplementary Figure 2), demonstrating the absence of protein and nucleic acid (Table 1). As presented in Supplementary Figure 3, ZSPP-1 shows an aggregated flake structure, stacked together with each other, small fragment structure and scattered distribution, and there are more gaps between fragments from the appearance and morphology.

**TABLE 1:** Chemical composition of ZSPP-1.

Parameters	Sugar content (%)	Uronic acid content (%)	Protein content (%)	Molecular weight (Da)	Sugar component (mol%)							
					
					Rha	Ara	Xyl	Man	Glc	Gal	GlcA	GalA
ZSPP-1	90.6 ± 3.6	12.31 ± 4.2	–	1.57 × 10^6^	–	4.3	9.8	22.8	20.7	36.8	2.9	2.7

### Homogeneity and molecular mass

The homogeneity was validated by elution, obtaining a single symmetrical peak at 8.37 min using high performance gel permeation chromatography (Supplementary Figure 4). Following are the calibration equations for carbohydrates with varying molecular weights: log M = −0.2798x + 8.5389 (*R*^2^ = 0.9964), M and x are the molecular weight and retention time of ZSPP-1, respectively; The molecular weight of ZSPP-1 is estimated to be 1.57 × 10^6^ Da (Table 1).

### Monosaccharide composition

According to GC-MS analysis, the monosaccharide composition of ZSPP-1 is illustrated in Table 1. The monosaccharide species were investigated in ZSPP-1 (seven types), indicating the structural complexity of ZSPP-1. As shown in Table 1, the ZSPP-1 was predominantly composed of galactose (36.8%), mannose (22.8%), and glucose (20.7%) in association with a small number of xylose (9.8%), arabinose (4.3%), glucuronic acid (2.9%), galacturonic acid (2.7%) units.

### Fourier transform infrared spectrometer analysis

**FIGURE 3:**
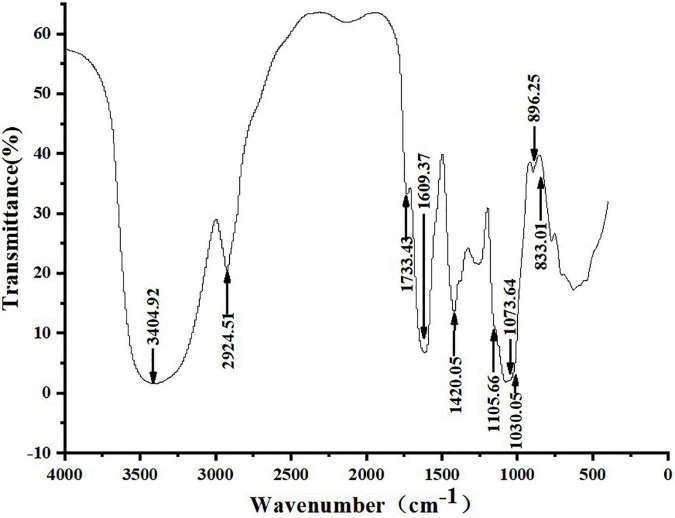
Fourier transform infrared spectrometer (FT-IR) spectrum of ZSPP-1.

Figure 3 shows that ZSPP-1’s infrared spectrum exhibited a typical polysaccharide characteristic band, a broad band in the range of 3,000–3,750 cm^–1^, and a strong absorption peak at 3,404.92 cm^–1^, which is the -OH stretching vibration signal peak (18); The weak absorption peak at 2,924.51 cm^–1^ was the C-H stretching vibration signal peak (19); The above two peaks were typical hydroxyl and alkyl groups of polysaccharides, denoting that the sample is polysaccharide (20). Moreover, infrared analysis showed that there was a weak absorption peak near 1,730 cm^–1^, and 1,733.43 cm^–1^ was the -COOH stretching vibration signal peak, indicating that the purified polysaccharide ZSPP-1 contained uronic acid (21). A strong absorption peak emerges at 1,609.37 cm^–1^, which is the stretching vibration of C = O, signifying the presence of -CHO; 1,420.42 cm^–1^ was the C-H variable angle vibration signal peak, and the above two peaks are also the infrared characteristic absorption peaks of polysaccharides. The band at approximately 1,000–1,200 cm^–1^ presents the existence of C-O-C and C-O-H bonds. The peaks at 1,105.66, 1,073.64, and 1,030.05 cm^–1^ are three signal peaks resulting from the stretching vibration of C-O bond and C-C bond in the sugar ring, which proves that ZSPP-1 contains pyran monosaccharide (22); The bands of 896.25 and 833.01 cm^–1^ proved that the pure polysaccharide ZSPP-1 existed simultaneously α- and β-glycosidic bond of configuration (23).

### Methylation analysis

To assess the chemical structure of glycosidic bonds in polysaccharides, methylation analysis is commonly employed. In this study, the methylation results were analyzed using the PMAA spectral standard database (complex carbohydrate research center, University of Georgia). Supplementary Figure 5 depicts the comparison of FT-IR spectra before and after ZSPP-1 methylation. Using methylation analysis, the glycosidic linkage and molar ratios of sugar residues in ZSPP-1 were determined. As shown in Table 2, the ZSPP-1 PMAA derivatives were measured to be 2,3,4,6-Me4-Man*p*, 2,3,5-Me3-Xyl*p*, 3,5-Me2-Xyl*p*, 2,3,6-Me3-Gal*p*, 2,3,4-Me3-Glc*p*, 2,3-Me2-Ara*p*, 2-Me-Glc*p*, and 3,4,6-Me3-Glc*p* with the molar of 21.5: 3.4: 5.66: 33.9: 11.1: 3.8: 6.74: 3.07. As shown by the monosaccharide composition of ZSPP-1, the total galactose content was the predominant fraction in ZSPP-1. Furthermore, the content of galactose and glucose increased after uronic acid reduction, signifying the content of galactose and glucose increased after methylation (24). This result suggested that ZSPP-1 mainly contained seven linkages: Man*p*-(1→, →2)-Xyl*p*-(1→, →4)-Gal*p*-(1→, →6)-Glc*p*-(1→, →4)-Ara*p*-(1→, →3,4,6)-Glc*p*-(1→, →2)-Glc*p*-(1→, and Xyl*p*-(1→ respectively, in which →4)-Gal*p*/Gal*p*A-(1→ might be the main linkage of ZSPP-1.

**TABLE 2:** Methylation analysis data for ZSPP-1.

Retention time	Methylated sugars	Mass fragments (m/z)	Molar ratio	Type of linkages
29.409	2,3,4,6-Me4-Man*p*	43, 59, 71, 87, 101, 113, 129, 145, 157, 162, 205	21.5	Man*p*-(1→
29.641	2,3,5-Me3-Xyl*p*	43, 59, 73, 87, 101, 115, 129, 146, 157	3.4	Xyl*p*-(1→
32.022	3,5-Me2-Xyl*p*	43, 59, 74, 85, 99, 118, 130, 142, 160, 173,	5.66	→2)-Xyl*p*-(1→
32.254	2,3,6-Me3-Gal*p*	43, 59, 71, 87, 99, 118, 131, 142, 157, 173, 203	33.9	→4)-Gal*p/*Gal*p*A-(1→
33.184	2,3,4-Me3-Glc*p*	43, 59, 71, 87, 99, 118, 129, 143, 159, 173, 189	11.1	→6)-Glc*p*-(1→
33.610	2,3-Me2-Ara*p*	43, 59, 71, 87, 101, 118, 129, 142, 162, 173	3.8	→4)-Ara*p*-(1→
34.616	2-Me-Glc*p*	34, 59, 87, 97, 118, 139, 160, 171, 202, 231	6.74	→3,4,6)-Glc*p*-(1→
35.275	3,4,6-Me3-Glc*p*	34, 59, 74, 87, 113, 129, 160, 190, 234	3.07	→2)-Glc*p*-(1→

### NMR analysis

**FIGURE 4:**
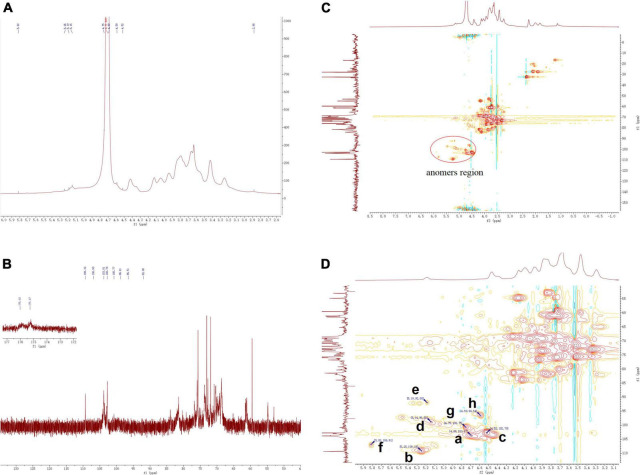
**(A)** The 1H NMR, **(B)** 13C NMR, and **(C,D)** HSQC spectra of ZSPP-1.

The structural features of ZSPP-1 were further identified by ^1^H, ^13^C, HSQC NMR spectral analysis at 600 MHz were investigated (Figures 4A–D). The entire assignment shifts of the ^1^H and ^13^C for ZSPP-1 were identified with reference to the previous literatures and then illustrated.

The chemical shifts in ^1^H NMR spectrum (Figure 4B) and HSQC spectra (Figures 4C,D) presented eight signals in the anomeric region at δ 4.69, δ 5.25, δ4.52, δ 5.14, δ 5.19, δ 5.82, δ 4.75, and δ 4.59 ppm. These eight anomeric protons were assigned to nine distinct glycosidic bond types. The chemical shifts in ^13^C NMR and HSQC spectra, these eight anomeric carbon signals appeared at δ 103.67, δ 109.18, δ 102.7, δ 98.80, δ 92.00, δ 106.91, δ 100.75, and δ 96.54 ppm, and all carbon chemical shifts were assigned to eight different types of glycosidic bonds. The eight detected sugar moieties were labeled as a, b, c, d, e, f, and h.

By combining the results of methylation analysis and literature data (25), the corresponding chemical shift at δ 106.91 ppm in the ^13^C NMR and HSQC spectra could be identified as β→4)-Ara*p*-(1→. Particularly, the ^13^C NMR signal at δ 175.27 and δ 176.02 ppm should be assigned to the carboxyl group of Glc*p*A and Gal*p*A, which indicated that ZSPP-1 was a novel acidic polysaccharide (26). The signals of ^13^C at δ 98.80 and δ 92.00 ppm assigned from the HSQC were inferred α→4)-Gal*p*A-(1→ and α→6)-Glc*p*A-(1→. As reported in the literature (27), when δ > 101 ppm, the signals belonged to the (1→2,3,4,6)-linked Gal and the (1→6,1→3,6)-linked Man. Therefore, the signals δ 102.7 and δ 103.67 ppm were inferred as β→4)-Gal*p*-(1→ and Man*p*-(1→ According to relevant references (28), the anomeric carbon signal peak of glucose residues were dispersed between δ 92 and δ 103 ppm (29). Therefore, the signal δ 92.00 and δ 100.75 ppm were inferred as α→3,4,6)-Glc*p*-(1→ and α→2)-Glc*p*-(1→. However, due to NMR signal peak of xylose residues were rarely reported in the literature, the corresponding chemical shifted at δ 109.18 ppm in ^13^C NMR and HSQC spectra could be inferred as xylose residues. This outcome requires additional discussion and clarity.

### ZSPP-1 administration altered the structure of the gut microbiota

To determine if ZSPP-1 can regulate or restore the imbalance of gut microbiota in obese mice, HFD mice were fed a ZSPP-1-supplemented diet for approximately 8 weeks, and their gut microbiota was studied.

As illustrated in the Venn diagram of OTU (Supplementary Figure 6), a total of 4,371 OTUs were obtained. In this study, the number of OTUs in NG, MG, LZG, MZG, HZG, and CG groups were 813, 747, 693, 725, 804, and 589, respectively. The composition similarity and overlap of OTUs among different treatment groups were studied. It was figured out that each group shared 471 OTUs. Meanwhile, 342, 276, 222, 254, 333, and 118 OTUs were unique to NG, MG, LZG, MZG, HZG, and CG groups, respectively. The increase in OTU abundance does not necessarily indicate a return to normal intestinal flora (30). As can be seen, however, the addition of ZSPP-1 altered the quantity of intestinal flora in HFD mice, and the abundance boosted with increasing dose.

**FIGURE 5:**
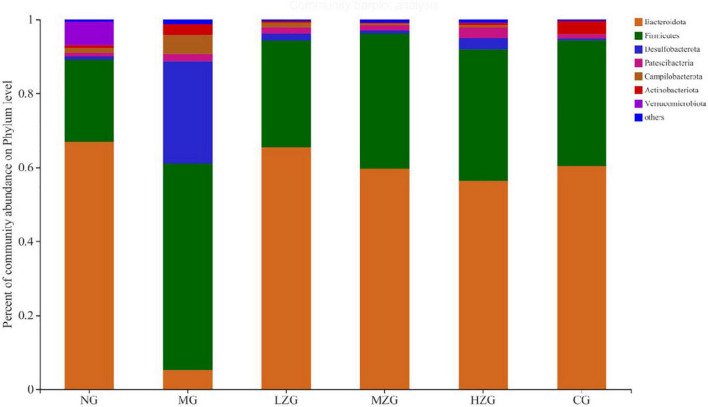
Gut microbial composition of different samples at the phylum level. The abscissa is the sample name, the ordinate is the proportion of the species in the sample, the columns with different colors represent different species, and the length of the columns represents the proportion of the species.

The analysis of the intestinal microbiota composition at the phylum level (Figure 5) exhibited that the gut microbiota of each treatment group consisted primarily of Bacteroidetes, Firmicutes, Desulfobacterota, Patescibacteria and Campylobacter. It is reported that the ratio of F/B value in intestinal microorganisms of obese mice will increase significantly (31). Compared with NG group, the F/B rate of MG group increased significantly; Besides, the F/B rate of LZG, MZG, HZG, and CG groups presented a downward trend compared with the NG group. Orlistat intake decreased the diversity and richness of intestinal microorganisms. Compared with the MG group, the proportions of Firmicutes and Bacteroidetes were further decreased by orlistat treatment, which reduced the abundance of obesity-associated bacteria Lachnospira.

These results are consistent with the findings of Ke et al. (32). Literature reports also confirmed that prebiotics can increase the relative abundance of Bacteroidetes and reduce the relative abundance of Firmicutes in the host intestine, so as to inhibit obesity (33). In this study, it was discovered that the relative abundance of Desulfobacterota and Campylobacter increased in the MG group, but decreased in the ZSPP-1 and positive medication treatment groups in comparison to the NG group.

**FIGURE 6:**
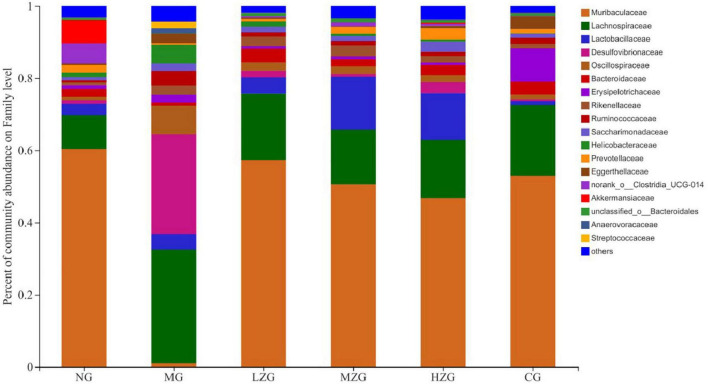
Gut microbial composition of different samples at the family level. The abscissa is the sample name, the ordinate is the proportion of the species in the sample, the columns with different colors represent different species, and the length of the columns represents the proportion of the species.

At the family level, *Muribaculaceae*, *Lachnospiraceae*, *Lactobacillus*, *Desulfovibrionaceae*, *Oscillospiraceae*, *Bacteroidaceae*, *Rikenellaceae*, and *Ruminococcaceae* were the main components of gut microbiota as shown in Figure 6. According to relevant literature reports, *Muribaculaceae*, *Bacteroidaceae*, and *Rikenellaceae* are mucin monosaccharide feeders. Many intestinal pathogens can utilize mucin monosaccharide as an essential nutrient in the intestine and compete with pathogens for these nutrients in order to maintain a healthy intestinal ecosystem (34). In this study, except for MG group, the relative abundance of *Muribaculaceae* and *Bacteroidaceae* in NG, LZG, MZG, HZG, and CG groups increased significantly, demonstrating that mucin monosaccharide seekers may form a competitive relationship with intestinal harmful bacteria. In addition, *Ruminococcaceae* is a potential diagnostic indicator of obesity and flora imbalance generated by HFD, whereas *Lachnospiraceae* is thought to be associated with liver inflammation (35). In this study, it was investigated that the abundance of *Lachnospiraceae* and *Ruminococcaceae* increased significantly in MG group, but decreased in ZSPP-1 and positive drug treatment group, which verified the view that mucin monosaccharide seekers formulated competitive correlation with intestinal harmful bacteria. LPS has been proven to be closely associated with the occurrence and development of chronic inflammation and metabolic disorders (36); *Desulfovibrionaceae* is a category of sulfate reducing bacteria, which can convert sulfate into hydrogen sulfide and also can destroy the integrity of intestinal barrier. Furthermore, *Desulfovibrionaceae* belongs to Gram-negative bacteria containing LPS. The results displayed that after ZSPP-1 intervention, the abundance of *Desulfovibrionaceae* decreased significantly in obese mice. The glucan components that make up ZSPP-1 [e.g., →6)-Glc*p*-(1→, →3,4,6)-Glc*p*-(1→, and →2)-Glc*p*-(1→] are expected to form prebiotics through the fermentation of intestinal flora, which possess a role in decreasing inflammatory cells and inflammatory mediators. By modulating the immune response in liver tissue, Neyrinck et al. (37) revealed that using fermentable laminarin glucan can protect rats from LPS-induced hepatotoxicity. Meanwhile, Li et al. (38) characterized the structure of Tuber sinense polysaccharide (TPS) and found that TPS has → 4)-D-Glc*p*-(1→, →4,6)-D-Glc*p*-(1→, and D-Glc*p*-(1→ residue; TPS and β-lactoglobulin binds to form a conjugate can increase some probiotics including *Lactobacillaceae*, inhibit harmful inflammatory reaction, and avoid intestinal flora disorder. This also confirmed that glucan-containing ZSPP-1 may increase the number of *Lactobacillaceae* in the intestines of obese mice. *Lactobacillaceae* is an essential probiotic for preventing and treating metabolic disorders such as obesity and diabetes mellitus (39, 40); *Prevotellaceae* is considered related to the synthesis of short chain fatty acids (SCFAs). In addition, the lack of SCFAs weakened its protective effect on intestinal mucosal barrier, which may render the increase of intestinal endotoxin level (41, 42); In this study, ZSPP-1 intervention could significantly enhance the abundance of *Lactobacillus* and *Prevotellaceae* in the intestinal microbial composition of obese mice. In conclusion, these findings demonstrate that ZSPP-1 positively regulates the imbalance of gut microbiota induced by obesity.

**FIGURE 7:**
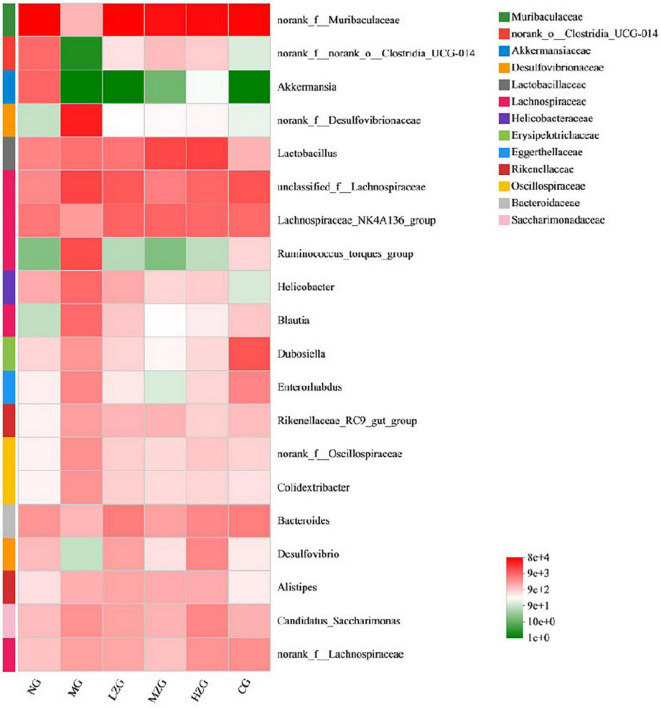
Heat map of gut microbial composition at the genus level. The abscissa is the group name, and the ordinate is the species name. The abundance changes of different species in the sample are displayed through the color gradient of the color block. The right side of the figure is the value represented by the color gradient.

Results as shown in Figure 7, at the genus level, the relative abundance of harmful bacteria (such as *Desulfovibrionaceae*, *Lachnospiraceae*, *Ruminococcus*, *Helicobacter*, *Oscillospiraceae* etc.) decreased significantly and the relative abundance of beneficial bacteria (such as *Akkermansia*, *Lactobacillus*, *Bacteroides*) increased significantly in LZG, MZG, HZG and CG groups compared with MG group. It was reported that *Desulfovibrionaceae*, *Lachnospiraceae*, *Ruminococcus*, *Helicobacter*, and *Oscillospiraceae* can render chronic inflammatory and metabolic illnesses (43). It has also been reported that squid ink polysaccharide (Sugar component: Fuc, GlcA, and GalN in a molar ratio of 1:1:1) decreases the abundance of harmful bacteria (such as *Ruminococcus*, *Bilophila*, *Oscillospira*, *Dorea*, *Mucispirillum*, etc.) which destroy the mucus layer on the colon surface and induce inflammatory diseases in the early stage (44). This suggests that ZSPP-1, comprised of seven monosaccharides GlcA, GalA, Ara, Xyl, Glc, Man, and Gal in a precise molar ratio of 1:1:1.5:3.6:7.6:8.4:13.6, may be the primary factor in reducing the number of hazardous bacteria. On the contrary, *Akkermansia*, *Lactobacillus*, and *Bacteroides et al*. can improve tissue inflammation resulted from obesity and boost host intestinal health (45). Polysaccharide molecular weight and monosaccharide composition have a crucial influence in the regulation of intestinal flora. Zhou et al. (46) assumed that ginseng polysaccharide (with molecular weight ranging from 1.00 to 1,308.98 kDa) made up of six monosaccharides (i.e., mannose, rhamnose, glucose, galactose, arabinose, and fucose) and one type of uronic acid (i.e., galacturonic acid) could restore the disturbed overall intestinal microbiota, especially promoting the growth of two main *Lactobacillus* and *Bacteroides*. Shang et al. (47) demonstrated that fucoidan, mostly consisting of fucose, glucuronic acid, and galactose, could ameliorate the metabolic syndrome brought on by HFD and increase the amount of *Akkermansia* bacteria in the intestinal microbiota of mice. Interestingly, this study discovered that the specific polysaccharide molecular weight (1,570 kDa) and monosaccharide composition of ZSPP-1 are similar to those reported in the literature, which may also be the key factor for ZSPP-1 to promote the growth of benign microorganisms conducive to the health of the host (e.g., *Akkermansia*, *Lactobacillus*, and *Bacteroides et al*.).

### ZSPP-1 administration recovered the original structure of intestinal microbiota

The visual circle diagram reflected the distribution proportion of dominant species in each group, as illustrated in Supplementary Figure 7. The proportion of Firmicutes and Bacteroidetes in the MG group has been grossly misadjusted in comparison to the NG group. The outcomes were comparable to those reported by Clarke et al. (4). Nonetheless, it can also be seen from Supplementary Figures 7B,C that the F/B value in the ZSPP-1 treatment group is no longer significant, demonstrating that Firmicutes and Bacteroidetes gradually returned to the normal level under the intervention of ZSPP-1.

**FIGURE 8:**
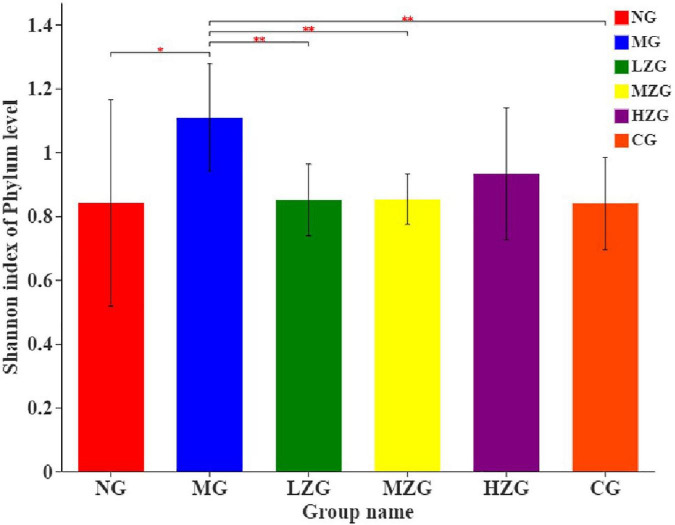
Alpha diversity analysis of the gut microbiota. The abscissa is the name of the group, and the ordinate is the average value of the index of each group. This figure shows the significant differences between the selected two groups of samples, and marks the two groups with significant differences. “*” means *P* < 0.05, “**” means *P* < 0.01.

Shannon index was applied to consider the richness and evenness of a community (48, 49). Through the assessment of alpha diversity abundance information of Shannon index at the phylum level in conjunction with the statistical *T*-test method, a significant difference between the two groups was identified (Figure 8). The results demonstrated that the MG group differed significantly from the LZG, MZG, CG, and NG groups; However, there was no significant difference between the ZSPP-1 treatment group and the NG group, stating that ZSPP-1 treatment of mice with an imbalanced gut microbiota restored the original bacterial structure.

**FIGURE 9:**
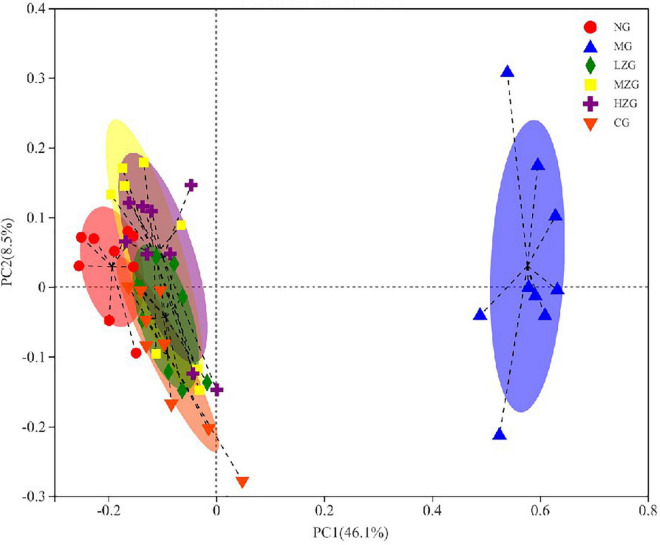
Beta diversity analysis of the gut microbiota (i.e., principal coordinates analysis). Points with different colors or shapes represent samples in different groups. The closer the two sample points are, the more similar the species composition of the two samples is.

Principal coordinates analysis (PCoA) is used to analyze the composition of different samples. Through studying the differences and distances of samples, the Multi group difference data were reflected on the two-dimensional coordinate map. In other words, the smaller the distance on the coordinate axis, the more similar the composition of the two samples, so it is possible to determine if the composition of the samples under identical conditions is similar. As exhibited in Figure 9, the intestinal microorganisms of NG group, CG group, and ZSPP-1 treated group mice differed significantly from those of MG group mice; The flora in LZG group and NG group had a more similar composition, yet such similarity did not rise as the ZSPP-1 intervention dose was increased. This suggests that ZSPP-1 restored the structure of gut microbiota without regard to dose. In addition, PCoA analysis demonstrated that the flora structure of the MZG and HZG groups was not strictly consistent with that of the NG group. It demonstrated that the adding of medium and high doses of ZSPP-1 not only restored the intestinal flora, but posed a novel regulatory effect on the intestinal flora’s composition.

Overall, in this study, through the changes of flora after different doses of ZSPP-1 intervention, it shows that ZSPP-1 regulates the structure of gut microbiota, improves the inflammation caused by obesity, and promotes intestinal health. Generally, the modulation of intestinal flora by polysaccharides is generally influenced by numerous factors. First, the chemical structure of polysaccharides is the foundation for the regulation of intestinal flora. By fermenting polysaccharides, intestinal flora may produce prebiotics, which can not only govern the structure of intestinal flora but also alter the host’s metabolism to achieve lipid-lowering and weight loss. The effects of polysaccharide molecular weight, composition, glycosidic bond type, substituent group, and spatial structure on intestinal flora and obesity have received limited investigation. The team of Sadia Kanwal and Yi Xin found *Dictyophora indusiata* mushroom polysaccharide with the main functional components of Glu (59.84%), Man (23.55%), and Gal (12.95%); Dip can reduce inflammatory response and alleviate HFD induced obesity by regulating intestinal integrity and intestinal microbial community (50–52). Therefore, there is justification to suppose that the effect of ZSPP-1 on the composition of intestinal flora in obese mice is mostly attributable to its unique functional components (36.8% galactose, 22.8% mannose, and 20% glucose).

## Conclusion

A diet high in sugar and fat can alter the structure of gut microbiota, resulting in obesity and chronic metabolic illnesses; nevertheless, plant polysaccharides can modulate gut microbiota. In this study, a polysaccharide was separated from *Z. striolatum*. Using various methodologies and instruments, the chemical structure of ZSPP-1 was determined. It was discovered that ZSPP-1 altered the structure of the gut microbiota of obese mice, stimulated the growth of intestinal beneficial bacteria, and assisted in restoring the imbalanced flora structure to its normal form. The aforementioned outcomes suggest that ZSPP-1 may be a potential drug for addressing HFD-induced obesity, which necessitates extensive research. Meanwhile, the analysis of chemical structure and the investigation of intestinal regulatory function provide a theoretical foundation for the discovery of the structure-function relationship, as well as the utilization and advancement of *Z. striolatum*.

## Data availability statement

The datasets presented in this study can be found in online repositories. The names of the repository/repositories and accession number(s) can be found below: https://submit.ncbi.nlm.nih.gov/subs/, accession number: PRJNA871034.

## Ethics statement

The animal study was reviewed and approved by Tianjin University of Science and Technology Experimental Animal Center.

## Author contributions

WJ: writing–original draft-lead and writing–review and editing-lead. YH: investigation-supporting, validation-supporting, and visualization-supporting. ZZ: writing–original draft-supporting and writing–review and editing-supporting. All authors contributed to the article and approved the submitted version.
