# Supplementary material for: Structural characteristics of polysaccharide from Zingiber striolatum and its effects on gut microbiota composition in obese mice
Source: Front Nutr. 2022 Oct 26;9:1012030. doi: 10.3389/fnut.2022.1012030 (PMC9643871; doi:10.3389/fnut.2022.1012030)
Supplement: Supplementary file 1 [file Table_1.DOCX]

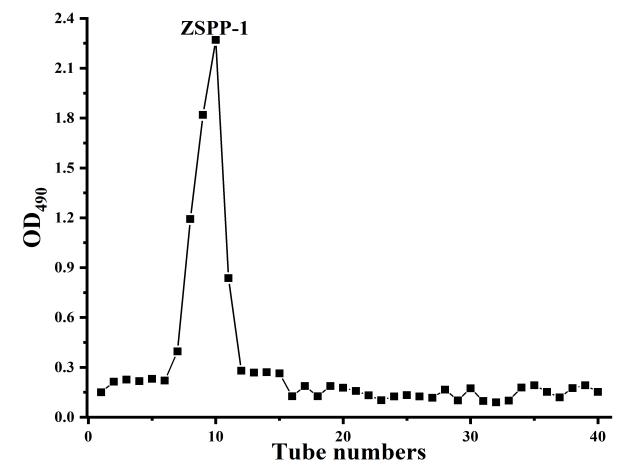


**Fig. S1.** Elution profile of ZSSP-1 on Sephadex G-150 gel column.


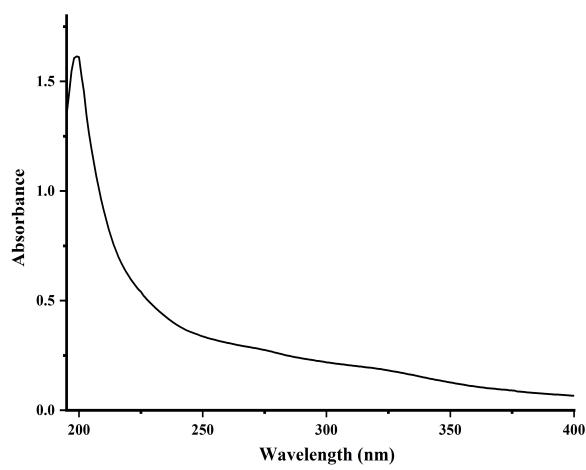


**Fig. S2.** UV–vis spectrum of ZSSP-1.


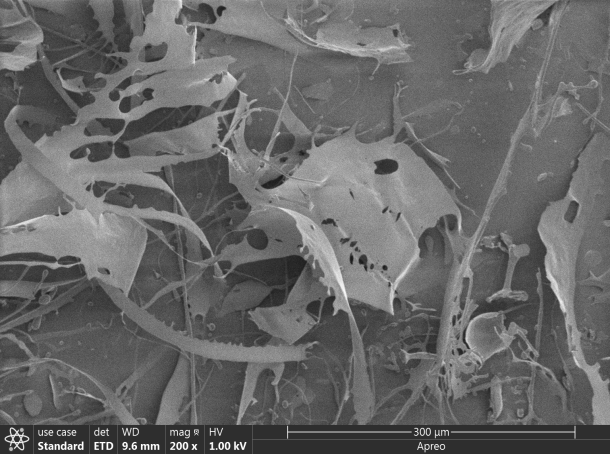


**Fig. S3.** SEM photographs of ZSSP-1（200×）.

**Fig. S4.** HPGPC chromatography of ZSPP-1.

Fig. S5. FT-IR spectra of ZSSP-1 (Before methylation) and methylation of ZSSP-1 (After methylation).


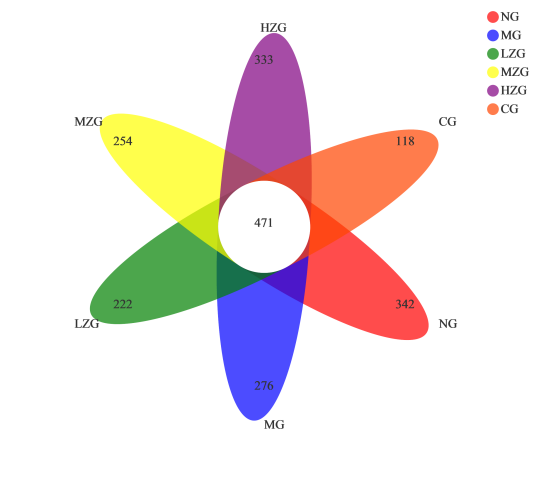


**Fig. S6** OTU Venen analyses of different samples.


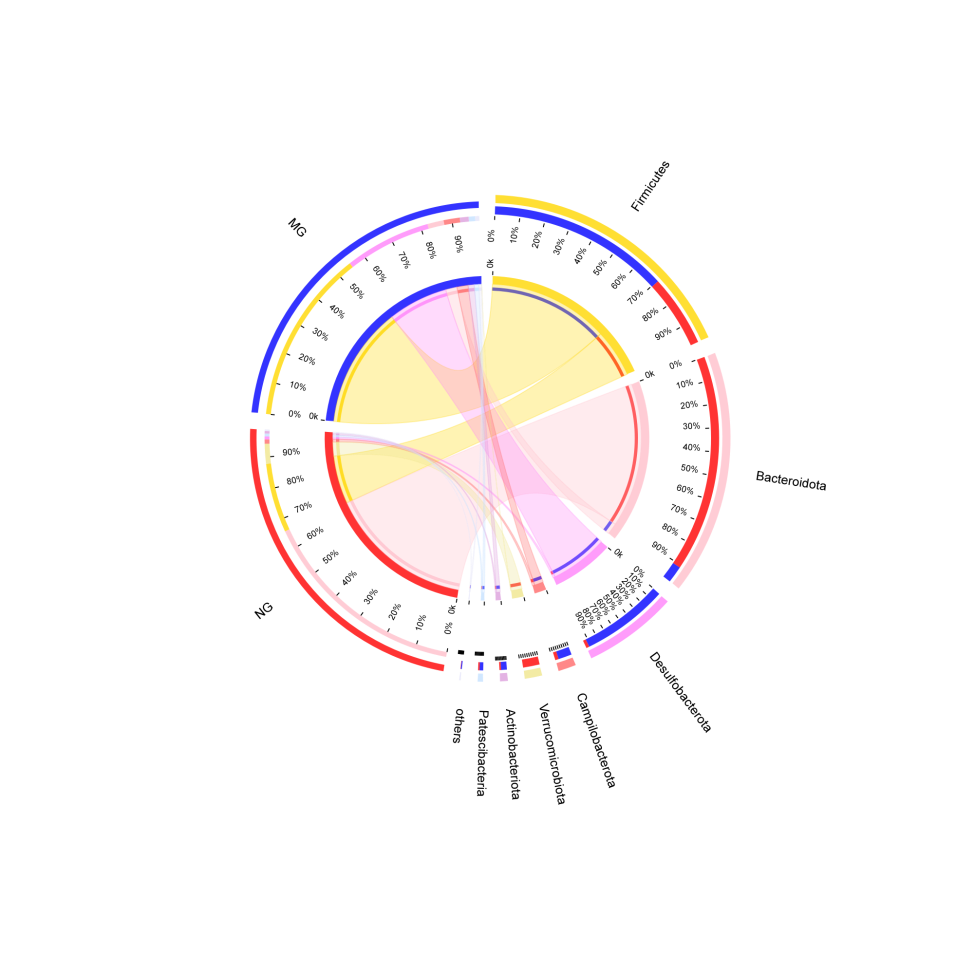

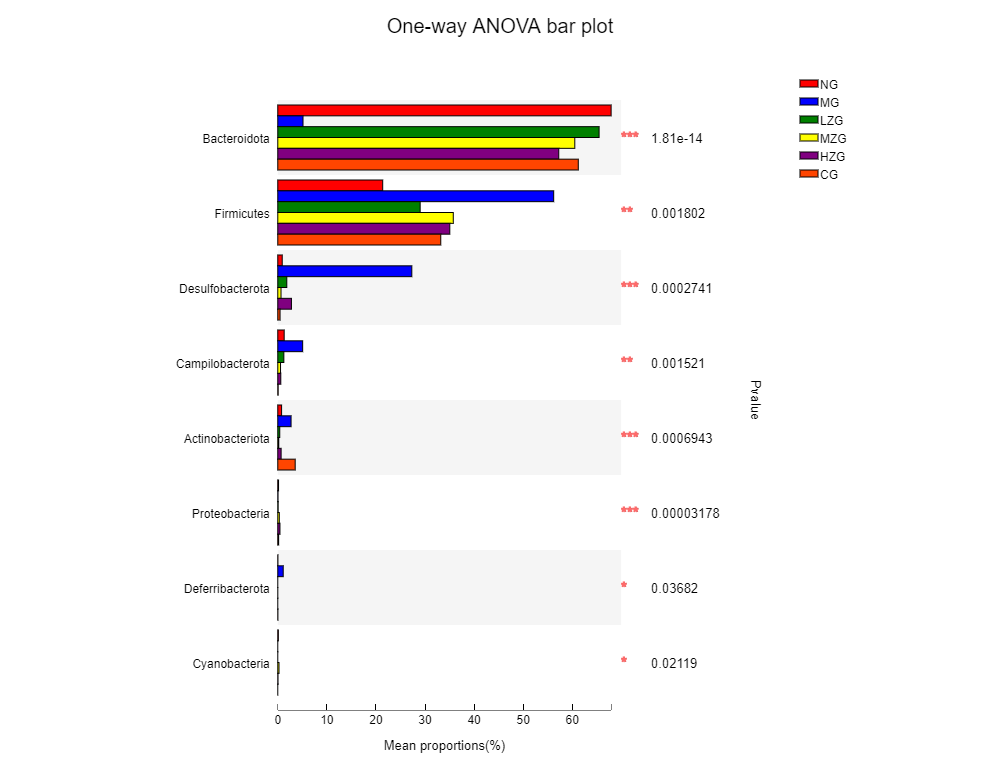


**B**

**A**


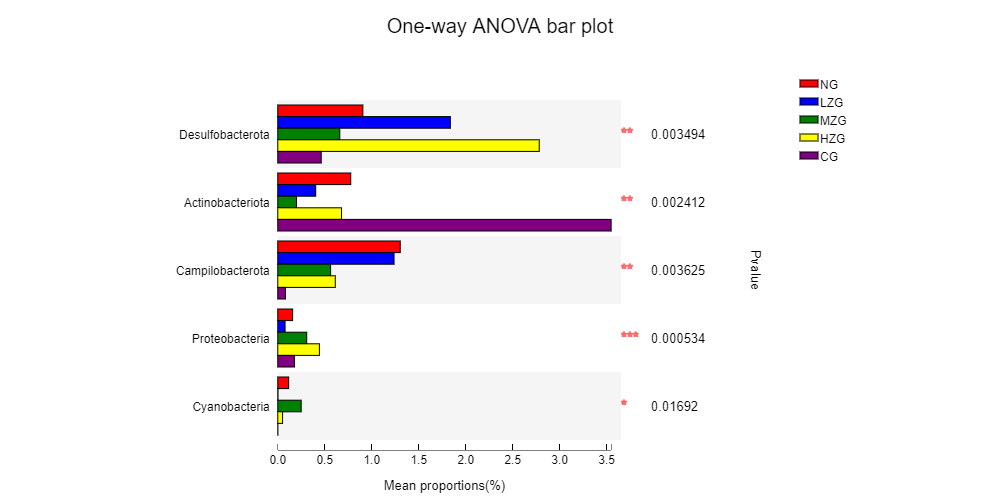


**C**

**Fig. S7** The visual circle chart reflects the distribution proportion of dominant species in NG group and MG group in various groups (A); Firmicutes and Bacteroidetes in MG group were significantly different from other groups (B); There was no significant difference in Firmicutes and Bacteroidetes between ZSPP-1 treatment group and NG group (C). (Note: the y-axis represents the species name at the phylum level, the x-axis represents the average relative abundance in different groups of species, and the columns with different colors represent different groups; The rightmost is p value, "*" means P ≤ 0.05, "* *" means P ≤ 0.01, "* * *" P ≤ 0.001.)
